# Salvianolic Acid B Prevents Iodinated Contrast Media-Induced Acute Renal Injury in Rats via the PI3K/Akt/Nrf2 Pathway

**DOI:** 10.1155/2016/7079487

**Published:** 2016-06-13

**Authors:** Liu Tongqiang, Liu Shaopeng, Yu Xiaofang, Song Nana, Xu Xialian, Hu Jiachang, Zhang Ting, Ding Xiaoqiang

**Affiliations:** ^1^Division of Nephrology, Zhongshan Hospital, Fudan University, Shanghai 200032, China; ^2^Division of Nephrology, The Affiliated Changzhou No. 2 Hospital of Nanjing Medical College, Changzhou, Jiangsu 213003, China; ^3^Shanghai Institute of Kidney and Dialysis, Shanghai 200032, China

## Abstract

Contrast-induced acute renal injury (CI-AKI) has become a common cause of hospital-acquired renal failure. However, the development of prophylaxis strategies and approved therapies for CI-AKI is limited. Salvianolic acid B (SB) can treat cardiovascular-related diseases. The aim of the present study was to assess the effect of SB on prevention of CI-AKI and explore its underlying mechanisms. We examined its effectiveness of preventing renal injury in a novel CI-AKI rat model. Compared with saline, intravenous SB pretreatment significantly attenuated elevations in serum creatinine and the histological changes of renal tubular injuries, reduced the number of apoptosis-positive tubular cells, activated Nrf2, and lowered the levels of renal oxidative stress induced by iodinated contrast media. The above renoprotection of SB was abolished by the PI3K inhibitor (wortmannin). In HK-2 cells, SB activated Nrf2 and decreased the levels of oxidative stress induced by hydrogen peroxide and subsequently improved cell viability. The above cytoprotection of SB was blocked by the PI3K inhibitor (wortmannin) or siNrf2. Thus, our results demonstrate that, due to its antioxidant properties, SB has the potential to effectively prevent CI-AKI via the PI3K/Akt/Nrf2 pathway.

## 1. Introduction

Contrast-induced acute renal injury (CI-AKI) is an important syndrome of acute renal failure occurring after the intravascular administration of radiographic contrast media (CM) in diagnostic and interventional procedures, which is defined as an increase of 25% or more or an absolute increase of 0.5 mg/dL or more in serum creatinine (Scr) from baseline value within 3 days after exposure to CM in the absence of any alternative causes [1]. CI-AKI is the third most common cause of acute renal failure in hospitalized patients [2] and is associated with replacement therapy, prolonged hospitalization, increased medical cost, and increased mortality [3, 4]. The present evidence indicates that the mechanisms of CI-AKI are thought to be a combination of the direct tubular toxicity of CM, renal medullary ischaemia, and generation of reactive oxygen species (ROS), in which ROS seem to represent the primary event in the pathogenesis of CI-AKI [4, 5]. Moreover, antioxidant-mediated protection of renal function with specific drugs provides indirect evidence that oxidative stress is considered to be involved in the pathogenesis of CI-AKI [6, 7].

Salvianolic acid B (SB) is one of the main components of Danshen (root of* Salvia miltiorrhiza*) [8], a widely used traditional Chinese medicine. Previous studies have shown that SB has antioxidative activity* in vivo* and* in vitro* [9, 10]. However, its mechanism for antioxidative damage is still not clear. It was reported that SB activated the PI3K/Akt signaling pathway [11] and induced nuclear factor erythroid 2-related factor 2 (Nrf2), heme oxygenase 1 (HO-1), and glutamate-l-cysteine ligase catalytic subunit (GCLC) expression, thereby protecting against APAP-induced liver injury and protecting dopaminergic neurons by an Nrf2-mediated action [9, 12]. Activation of the PI3K/Akt/Nrf2 pathway could protect against cell and organ injury through upregulation of antioxidant enzyme and phase II detoxification enzyme expression (e.g., HO-1; GCLC; and quinone oxidoreductase (NQO1)) [13].

Therefore, we hypothesized that SB could prevent AKI induced by CM because of its antioxidative effects. In the present study, the effects of SB on CI-AKI and its underlying mechanisms were investigated in an experimental model of CI-AKI in rats and human proximal tubule (HK-2) cells. Our results suggest that SB prevents CI-AKI by reducing oxidative stress through the PI3K/Akt/Nrf2 pathway.

## 2. Materials and Methods

### 2.1. Chemicals and Reagents

Iohexol (low-osmolarity nonionic CM, 350 mg iodine/mL, GE Healthcare, Shanghai, China), SB (purity >98.0%, kindly donated from Shanghai Green Valley Pharm. Co., Ltd.), wortmannin (Abcam Inc., Cambridge, MA, USA), sulforaphane (SFN, LKT Laboratories Inc., St. Paul, USA), Nrf2 siRNA (Santa Cruz, CA, USA), control siRNA (Santa Cruz, CA, USA), Opti-MEM I (Invitrogen, CA, USA), and Lipofectamine 2000 solution (Invitrogen, CA, USA) were used in this study.

### 2.2. Animals and Grouping

Male Sprague-Dawley rats (180–200 g) obtained from the Animal Center of Fudan University, Shanghai, China. The rats were acclimatized for 7 d before the start of study and handled in accordance with the institutional and national guidelines for animal research. A novel, reliable, and suitable CI-AKI model based on the 5/6 nephrectomy (NE) rat was established as in our previous reports [14, 15]. In brief, the CI-AKI model used 5/6 NE rats 6 weeks after an ablative surgery and was established by dehydration for 48 h, followed by administration of 10 mL/kg body weight (3.5 gI/kg) iohexol via the tail vein. All animals had* ad libitum* access to water and food after the injection.

#### 2.2.1. Part 1: The Role of SB in Renoprotection against Iohexol

Thirty-two rats with similar levels of renal function 6 weeks after 5/6 NE procedure were selected and randomly divided into the following four equal groups (*n* = 8 in each): (1) saline group: administered vehicle and 10 mL/kg 0.9% saline; (2) SB + saline group: administered 50 mg/kg SB and 10 mL/kg 0.9% saline; (3) CM group: administered vehicle and 10 mL/kg iohexol; and (4) SB + CM group: administered 50 mg/kg SB and 10 mL/kg iohexol. Intravenous injection of SB and vehicle was performed 5 min prior to the intravenous injection of saline or iohexol. Scr, blood urea nitrogen (BUN), and tissue morphology were assessed at 24 h after the last injection.

#### 2.2.2. Part 2: The Role of the PI3K/Akt/Nrf2 Pathway in Renoprotection Induced by SB

Eighty rats with similar levels of renal function 6 weeks after the ablative surgery were selected and randomly divided into the following six equal groups (the CM and SB (or vehicle) were administered as described above): (1) CM group; (2) SB + CM group; (3) Wort + CM group: treated with wortmannin (15 *μ*g/kg) [15], vehicle, and iohexol (10 mL/kg); and (4) Wort + SB + CM group: treated with wortmannin (15 *μ*g/kg), SB, and iohexol (10 mL/kg); and (5) SFN + CM group: treated with SFN (10 mg/kg) [16] and iohexol (10 mL/kg). Intravenous injection of the pharmacological inhibitors, vehicle, or SFN was performed 5 min prior to the SB or iohexol. The rats in each group were randomly divided into two equal subgroups (*n* = 8 in each). Rats of one subgroup were used for the detection of p-Akt/Akt and Nrf2 by Western blotting at 3 h after the last injection. This time point was selected because a preliminary experiment showed that the SB-induced p-Akt and nuclear-Nrf2 levels in kidney tissue peaked at 3 h and were maintained for 24 h in CI-AKI rats. The rats in the other subgroup were used for the detection of Scr, and haematoxylin and eosin (HE) staining and terminal deoxynucleotidyl transferase-mediated dUTP nick-end labeling (TUNEL) analysis of the kidneys tissue was performed at 24 h after the last injection.

### 2.3. Cell Culture and Treatment

HK-2 cells (American Type Culture Center, Manassas, Virginia), a human proximal tubular cell line, were cultured at 37°C in 5% humidified CO_2_ in Dulbecco's modified Eagle's medium supplemented with 10% fetal calf serum. Our preliminary study showed that the H_2_O_2_-induced ROS production peaked at 3 h and subsequently decreased cell viability at 12 h. Therefore, we evaluated cellular ROS levels at 3 h and cell viability at 24 h after treatment with H_2_O_2_. Specific targeted siRNA (siNrf2) was used in this part. Our preliminary study showed that the expression of nuclear-Nrf2 was decreased to approximately 20% of that in the control siRNA-transfected HK-2 cells.

The groups were as follows: (1) control group: the cells were treated with phosphate-buffered saline (PBS); (2) H_2_O_2_ group: cells were treated with 250 mM H_2_O_2_ for 3 h or 24 h; (3) SB group: cells were treated with 50 *μ*M SB for 1 h; (4) SB + H_2_O_2_ group: cells were treated with 50 *μ*M SB for 1 h and then treated with 250 mM H_2_O_2_ for 3 h or 24 h; (5) Wort group: cells were treated with an inhibitor (wortmannin, 10 *μ*M) for 1 h; (6) Wort + SB + H_2_O_2_ group: cells were treated with an inhibitor (wortmannin, 10 *μ*M) and 50 *μ*M SB for 1 h and then treated with 250 mM H_2_O_2_ for 3 h or 24 h; (7) siCTRL + SB + H_2_O_2_ group: control siRNA were dissolved separately in Opti-MEM I. After 10 min of equilibration at room temperature, each RNA solution was combined with the respective volume of the Lipofectamine 2000 solution, mixed gently, and allowed to form siRNA liposomes for 20 min. The primary cultured cells were transfected with the transfection mixture in antibiotic-free cell culture medium for 6 h; then the cells were treated with 50 *μ*M SB for 1 h and finally treated with 250 mM H_2_O_2_ for 3 h or 24 h; and (8) siNrf2 + SB + H_2_O_2_ group: cells were treated with Nrf2 siRNA, SB, and H_2_O_2_ as above protocol. Vehicle also was performed according to the experimental requirements.

### 2.4. Measurement of Cellular ROS Levels

CM-H2DCFCA, a ROS sensitive fluorescent dye, was used to measure ROS levels. To evaluate the effect of SB on H_2_O_2_-induced ROS production, HK-2 cells were cultured in 96-well plates. 5 mM CM-H2DCFCA was added to each well and incubated for 30 min in Dulbecco's PBS. Fluorescence intensities were measured at an excitation wavelength of 485 nm and an emission wavelength of 535 nm using a microplate fluorescence reader.

### 2.5. Measurement of HK-2 Cell Viability

HK-2 cell viability was assessed using a CCK-8 assay kit (Beyotime Institute of Biotechnology, Shanghai, China). Cells were cultured in 96-well plates. Approximately 10 *μ*L of CCK-8 solution was added to each well and incubated for 4 h at 37°C. Fluorescence intensities were measured at an excitation wavelength of 485 nm and an emission wavelength of 535 nm using a microplate fluorescence reader. The viability of HK-2 cells was quantified by fluorescence intensities using a spectrophotometer (Model 680 Microplate Reader, Berkeley, California).

### 2.6. Detection of Rat Serum Biomarkers

Approximately 0.5 mL of blood was collected from the jugular vein or the abdominal aorta. The blood was centrifuged at 2000 g for 10 min to obtain the serum. The Scr and BUN concentrations were determined using a Hitachi 7060 chemistry analyzer by a Jaffe method.

### 2.7. HE Staining

The kidney specimens were fixed in 4% formalin for 24 h and embedded in paraffin. Three *μ*m thick tissue sections were cut and stained with HE for morphologic analysis. A pathologist blinded to the study protocol analyzed the sections using a light microscope (Leica DM 6000 B; Leica Microsystems, Wetzlar, Germany). For semiquantitative analysis of morphological changes, we randomly selected 10 high-magnification (×200) fields of the cortex and the outer stripe of the outer medulla. The extent of foamy degeneration and detachment of tubular cells was graded with an arbitrary score of 0–4 as follows [14, 15]: no injury (0); mild: 0–25% (1); moderate: 25–50% (2); severe: 50–75% (3); and very severe: 75–100% (4).

### 2.8. TUNEL Assay

For TUNEL staining, 3 *μ*m paraffinized sections of the renal corticomedullary boundary zone were performed using a commercial kit (*In Situ* Cell Death Detection Kit; Roche, Basel, Switzerland) in accordance with the manufacturer's instructions. The sections were fixed in acetone for 10 min, washed in PBS, and immersed in a solution of 3% H_2_O_2_ for endogenous peroxidase blocking. Incubation with the TUNEL reaction mixture was then performed for 60 min. TUNEL-positive cells were shown with 3,3′-diaminobenzidine; sections were counterstained with hematoxylin. One hundred cells and the percentage of TUNEL-positive apoptotic cells were counted in five different fields (×200) per tissue slide under a light microscope by a pathologist in a blinded fashion.

### 2.9. Total and Nuclear Protein Extraction

The renal tissue or cultured cells were divided into two equal portions. One half was prepared in ice-cold lysis buffer (50 mM Tris-HCL pH 6.8, 150 mM NaCl, 5 mM EDTA, 0.5% sodium deoxycholate, 0.5% NP-40, and a protease inhibitor cocktail) using a homogenizer on ice. After centrifuging at 12,000 g for 15 min at 4°C, the supernatant containing the total protein extract was collected. The other half was prepared in an ice-cold hypotonic buffer (containing protease and phosphatase inhibitors, DTT, and detergent) using a homogenizer and centrifuged at 14,000 g for 30 s at 4°C. The nuclear pellet was resuspended in 50 *μ*L of complete lysis buffer, incubated on ice for 30 min, and centrifuged at 14,000 g for 10 min at 4°C. The supernatant containing the nuclear protein extract was collected, and the protein concentrations were determined using a BCA Protein Assay Reagent Kit (Beyotime Institute of Biotechnology, Shanghai, China).

### 2.10. Western Blot Assay

Equal amounts of proteins (40 *μ*g total protein or 80 *μ*g nuclear protein per lane) were loaded onto a 12.5% gradient Tris-HCl SDS polyacrylamide gel and then transferred to a PVDF membrane. Nonspecific binding to the membrane was blocked for 1 h at room temperature with 5% nonfat milk in 1 × TBS, followed by incubation with primary antibodies against total Akt (rabbit monoclonal 1 : 1000; Cell Signaling Technology, Danvers, MA), p-Akt (Ser473, rabbit monoclonal 1 : 2,000; Cell Signaling Technology), Nrf2 (mouse monoclonal 1 : 1000; Abcam Inc., Cambridge, MA, USA), heme oxygenase 1 (HO-1, mouse monoclonal 1 : 500; Abcam), *β*-actin (mouse monoclonal polyclonal 1 : 1,000; Abcam), or Histone H3 (mouse monoclonal polyclonal 1 : 500; Abcam) overnight at 4°C. After washing with TBST three times, membranes were incubated with horseradish peroxidase-conjugated rabbit or goat secondary antibody (1 : 10000 dilution; Kang Chen Biotechnology, Guangzhou, China) for 1 h at room temperature, followed by three washes for 10 min each. Blots were developed using enhanced chemiluminescent reagents (Thermo Fisher Scientific, Pittsburgh, PA, USA) and target band density was scanned using a LAS-3000 detection system. Image J software was used to analyze band intensities. The results were normalized to the protein levels of *β*-actin or Histone H3.

### 2.11. Immunohistochemical Staining for Nrf2 and the Oxidized Derivative (8-Hydroxydeoxyguanosine, 8-OHdG)

Immunohistochemical staining was performed on 3 *μ*m paraffinized sections. The samples were cleared in xylene and rehydrated in a series of ethanol washes. Endogenous peroxidase activity was inhibited with 3% H_2_O_2_ for 10 min and then treated with normal goat serum (1 : 20) for 20 min. Next, the samples were incubated with anti-Nrf2 antibody (mouse monoclonal 1 : 200; Abcam) or anti-8-OHdG antibody (mouse monoclonal 1 : 100; Abcam) at 4°C overnight. The sections were then incubated with a horseradish peroxidase-conjugated secondary antibody (anti-mouse IgG). After rinsing three times with PBS, the sections were stained with 3,3′-diaminobenzidine and then counterstained with hematoxylin. The stained specimens were assessed under a light microscope by a pathologist in a blinded fashion. We randomly selected five high-magnification (×200) fields of the renal corticomedullary boundary zone. The specimens were scored according to the percentage of Nrf2 and 8-OHdG positive cells.

### 2.12. Lipid Peroxidation/ROS Production

Malondialdehyde (MDA) is a naturally occurring product of lipid peroxidation and an indicator of ROS production. MDA cannot cover all unsaturated aldehydes and ketones produced by oxidative damage and lipid peroxidation. Nonlipid TBARS may be present in the sample. Therefore, MDA is not specific to lipid peroxidation. However, it is is widely used and is still a good indicator of oxidative stress. The dissected kidneys were immediately rinsed in ice-cold PBS. Tissues were homogenized in 10% 150 mM phosphate buffer (pH 7.4) (1/10 w/v). The homogenate was centrifuged at 6000 g for 10 min at 4°C. The total protein level in the supernatant was measured using a BCA Protein Assay Reagent Kit (Beyotime Institute of Biotechnology, Shanghai, China). The supernatant of the renal cortical homogenate was used to estimate lipid peroxidation according to the manufacturer's protocol (TBARS Assay Kit; Cayman Chemical Company, Ann Arbor, Michigan, USA). The level of lipid peroxides was expressed as nmol of MDA/g of kidney.

### 2.13. Statistical Analysis

Data are expressed as the means ± SD. Data were analyzed by one-way ANOVA with Tukey's multiple comparison (parametric tests) or Kruskal-Wallis test with Dunns' multiple comparison (nonparametric tests). Statistical significance of difference was defined as a *p* value < 0.05.

## 3. Results

### 3.1. Renoprotective Effect of SB on Contrast-Induced Nephropathy

**Figure 1:**
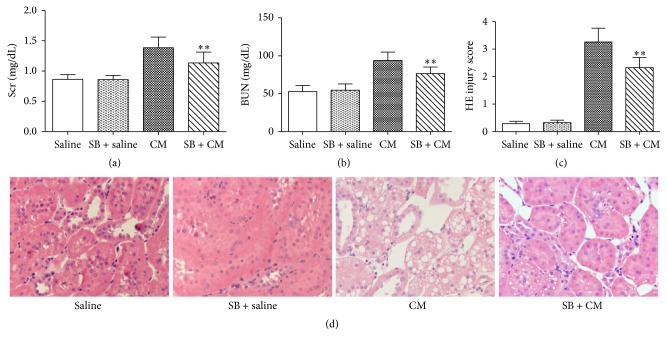
Protective effects of SB against CI-AKI. SB (50 mg/kg) ameliorated CM-induced increase in (a) the Scr level, (b) the BUN level, and (c) histologic injury scoring at 24 h after the intravenous injection of 10 mL/kg iohexol (350). (d) Representative histological image of kidney from representative photomicrographs of tubular cell injury in rat kidney tissue sections. SB significantly attenuated the renal tubular injury at 24 h after the injection of iohexol. Original magnification: ×200. HE staining. ^*∗∗*^ *p* < 0.01 versus the CM group; *n* = 8. The values shown are the mean ± SD.

The rat in the CM group exhibited elevations in Scr and BUN 24 h after iohexol injection (Figures 1(a) and 1(b)). The levels of Scr and BUN in the SB + CM group were markedly decreased compared with the CM group (Scr, 1.13 ± 0.18 mg/dL versus 1.39 ± 0.18 mg/dL; *p* < 0.01; BUN, 76.5 ± 8.7 mg/dL versus 93.9 ± 11.1 mg/dL; *p* < 0.01). The CM group showed severe tubular damage (detachment and foamy degeneration of tubular cells). The animals in the SB + CM groups showed significantly lower histologic injury scores in terms of tubular epithelium degeneration than those in the CM group (histologic scoring, 2.32 ± 0.37 versus 3.26 ± 0.50; *p* < 0.01) (Figures 1(c) and 1(d)).

### 3.2. SB-Mediated Renoprotection against Iohexol-Induced Injury through the PI3K/Akt/Nrf2 Pathway in Rats

**Figure 2:**
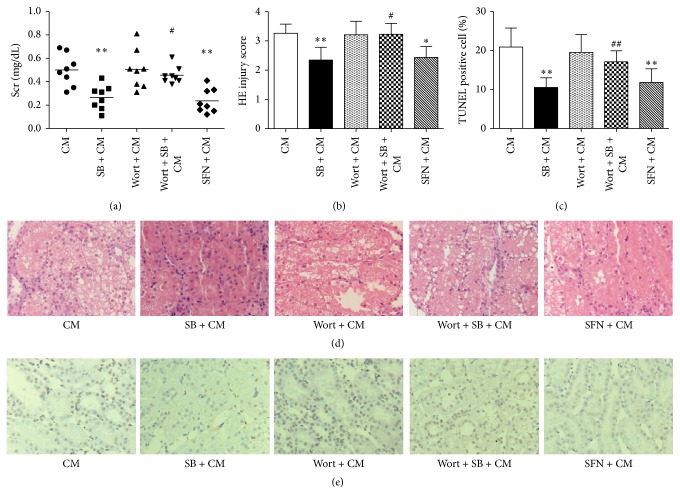
SB-induced renoprotection against CI-AKI involves the PI3K/Akt/Nrf2 pathway. (a) A marked reduction in ΔScr was induced by SB in the rats 24 h after intravenous injection of 10 mL/kg iohexol (350). This effect of SB was abolished by treatment with a specific inhibitor of PI3K (wortmannin) and an activator of Nrf2 (SFN) could mimic the effect of SB. (b, d) Representative photomicrographs of tubular cell injury in rat kidney tissue sections and quantitative analysis of histologic scoring (HE staining). SB significantly attenuated renal tubular injury 24 h after injection of iohexol. This effect of SB was abolished by treatment with wortmannin, and SFN could mimic the effect of SB. (c, e) Representative photomicrographs of immunohistochemical staining for TUNEL in renal sections and quantitative analysis of TUNEL-positive cells. The TUNEL-positive staining was localized in nuclei. SB significantly decreased the number of TUNEL-positive cells in rat kidney tissue sections 24 h after the injection of iohexol. This effect of SB was inhibited by treatment with wortmannin and SFN could mimic the effect of SB. Original magnification (d, e): ×200. ^*∗*^ *p* < 0.05 and ^*∗∗*^ *p* < 0.01 versus the CM group; ^#^ *p* < 0.05 and ^##^ *p* < 0.01 versus the SB + CM group; *n* = 8. The values shown are the mean ± SD.

To explore whether the PI3K/Akt/Nrf2 pathway is involved in the renoprotection provided by SB, a specific inhibitor of PI3K (wortmannin) and an activator of Nrf2 (sulforaphane) [17] were used in this experiment. Scr levels in the Wort + SB + CM group were also significantly increased in comparison with those in the SB + CM group (ΔScr, 0.46 ± 0.07 mg/dL versus 0.27 ± 0.10 mg/dL; *p* < 0.01) (Figure 2(a)). Scr levels in the SFN + CM group were not significantly different from those in the SB + CM group (ΔScr, 0.24 ± 0.10 mg/dL versus 0.27 ± 0.10 mg/dL; *p* > 0.01) (Figure 2(a)). Rats in the Wort + SB + CM group showed severe renal histological alterations 24 h after the iohexol injections compared with the SB + CM group (histologic scoring, 3.23 ± 0.38 versus 2.35 ± 0.43; *p* < 0.05) (Figures 2(b) and 2(d)). The renal histological alterations in rats in the SFN + CM group were not significantly different from that of the SB + CM group (2.44 ± 0.37 versus 2.35 ± 0.43; *p* > 0.05) (Figures 2(b) and 2(d)). TUNEL staining was performed to evaluate the levels of apoptosis in renal cells. The number of TUNEL-positive cells in the Wort + SB + CM group was markedly increased compared with the the SB + CM group (percentage of TUNEL-positive cells, 17.13 ± 2.82 versus 10.53 ± 2.47, *p* < 0.01) (Figures 2(c) and 2(e)). The number of TUNEL-positive cells in the renal sections in the SFN + CM group was not significantly different from the SB + CM group (11.83 ± 3.49 versus 10.53 ± 2.47, *p* > 0.05) (Figures 2(c) and 2(e)).

### 3.3. SB Reduced the Levels of Oxidative Stress, through Activation of Nrf2/HO-1, Which Was Abolished by Wortmannin, and Sulforaphane Mimicked This Effect of SB

SB is reported to exert antioxidative and organ-protective effects. To investigate whether SB decreases oxidative stress, the level of an oxidized nucleotide derivative (8-OhdG) in kidney sections and the amount of the lipid peroxidation final reaction substance (MDA) in the supernatants of renal cortical homogenates were determined.

**Figure 3:**
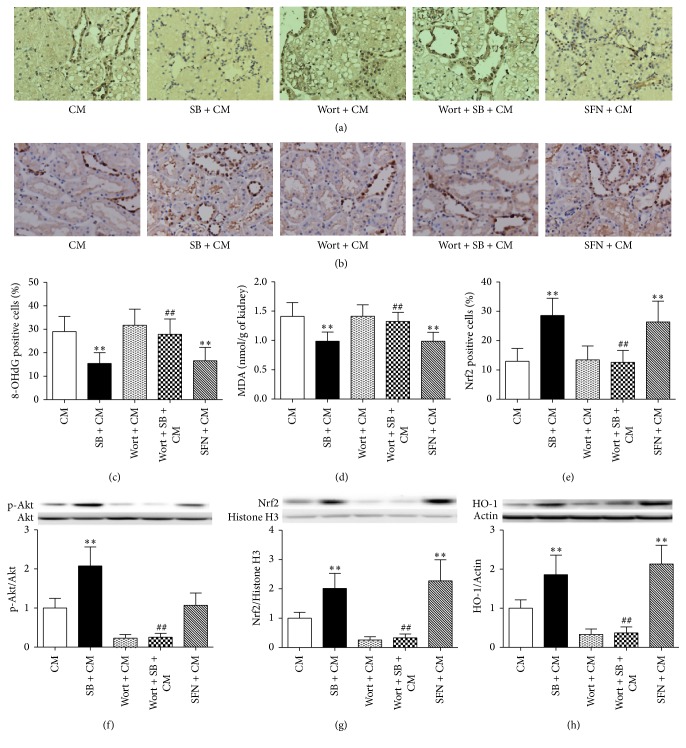
SB decreased the levels of oxidative stress induced by iohexol through the Akt/GSK-3*β*/Nrf2 pathway. (a, c) Representative photomicrographs of immunohistochemical staining for the redox product oxidized derivative, 8-OHdG, in renal sections and quantitative analysis of 8-OHdG-positive cells. The 8-OHdG-positive staining was localized in nuclei. (b, e) Representative photomicrographs of immunostaining for Nrf2 in the renal sections and quantitative analysis of the Nrf2-positive cells. The extent of staining for Nrf2 in the nuclear fraction represents their activity. (d) MDA concentrations in the renal tissues. SB resulted in a marked reduction in the number of 8-OHdG-positive cells and the MDA concentrations and a marked increase in the number of Nrf2-positive cells in renal tissues 24 h after iohexol injection. These effects were inhibited by treatment with wortmannin, and SFN could mimic the effects of SB. (f, g) Representative immunoblots and statistical data of (f) phosphorylated and total Akt (56 KDa), (g) nuclear-Nrf2 (68 KDa), and (h) HO-1 (32K Da) in the renal lysates from rats 3 h after the injection of iohexol. SB could activate Nrf2/HO-1 by inducing the phosphorylation of Akt, which was inhibited by wortmannin. SFN could activate Nrf2/HO-1. Note that SB protected the kidneys from oxidative stress through the PI3K/Akt/Nrf2 pathway. Original magnification (a, b): ×200. ^*∗∗*^ *p* < 0.01 versus the CM group and ^##^ *p* < 0.01 versus the SB + CM group; *n* = 8. The values shown are the mean ± SD.

The kidney tissue sections were taken in order to determine the immunohistological staining for 8-OhdG. Immunohistochemical staining showed that the 8-OhdG was expressed in the cell nucleus of kidney cell. The number of 8-OhdG-positive cells in the SB + CM group was significantly lower than those in the CM group (percentage of 8-OhdG-positive cells, 15.45 ± 4.63 versus 29.03 ± 6.45, *p* < 0.01) (Figures 3(a) and 3(c)). Furthermore, the levels of MDA in the kidneys of the SB + CM group were significantly lower than those in the CM group (0.99 ± 0.16 versus 1.41 ± 0.23, *p* < 0.01) (Figure 3(d)).

To further explore the antioxidative effects of Nrf2, sulforaphane was used in this experiment. We found that sulforaphane decreased the levels of oxidative stress (mimicking the effects of SB) (Figures 3(a), 3(c), and 3(d)). In addition, a specific inhibitor of PI3K (wortmannin) abolished the antioxidative effects of SB (Figures 3(a), 3(c), and 3(d)).

Only in the nuclear fraction, Nrf2 can exert antioxidative effects. Immunohistochemical staining showed that the numbers of Nrf2-positive cells were more in the SB + CM group and SFN + CM group than in the CM group (percentage of Nrf2-positive cells, 28.58 ± 5.92 versus 12.93 ± 4.42, *p* < 0.01; 26.40 ± 7.10 versus 12.93 ± 4.42, *p* < 0.01) (Figures 3(c) and 3(e)). Western blot analysis showed a significant increase in the levels of p-Akt, nuclear-Nrf2, and HO-1 in the SB + CM group compared with the CM group (p-Akt/Akt: 2.08 ± 0.48 versus 1.00 ± 0.25, *p* < 0.01; Nrf2: 2.02 ± 0.51 versus 1.00 ± 0.20, *p* < 0.01; HO-1/Actin: 1.86 ± 0.50 versus 1.00 ± 0.21, *p* < 0.01) (Figures 3(f), 3(g), and 3(h)). There was a significant increase in the levels of nuclear-Nrf2 and HO-1 in the SFN + CM group compared with the CM group (Nrf2: 2.27 ± 0.72 versus 1.00  ±  0.20, *p* < 0.01; HO-1/Actin: 2.13  ±  0.48 versus 1.00 ± 0.21, *p* < 0.01). There was no significant difference in levels of p-Akt between the SFN + CM group and the CM group (1.07 ± 0.31 versus 1.00 ± 0.25, *p* > 0.05) (Figures 3(f) and 3(g)). However, a specific inhibitor of PI3K (wortmannin) decreased the expression of p-Akt, nuclear-Nrf2, and HO-1 (Figures 3(f), 3(g), and 3(h)). The above results showed that SB exerted antioxidative effects through the PI3K/Akt/Nrf2 pathway.

### 3.4. Effect of SB on Cellular ROS Levels and Cell Viability Induced by H_2_O_2_ in HK-2 Cells

**Figure 4:**
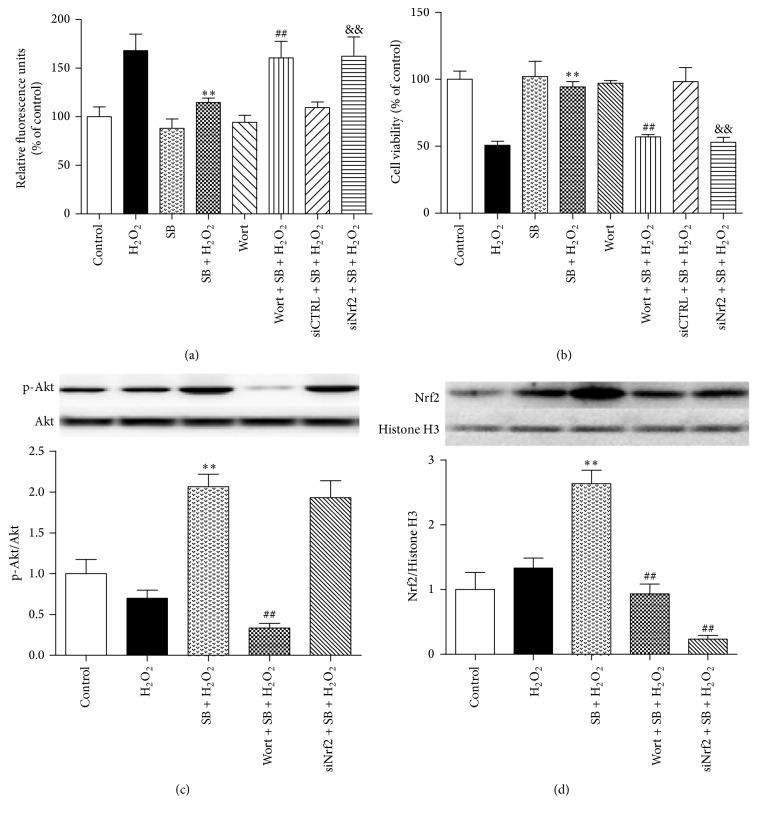
Effects of SB on ROS levels and the viability of renal tubular epithelial cells injured with H_2_O_2_. (a) HK-2 cells were incubated with SB (50 *μ*M) or vehicle for 1 h and then incubated with 250 mM H_2_O_2_ or vehicle for 3 h. Fluorescence intensity was measured at an excitation wavelength of 485 nm and an emission wavelength of 535 nm. (b) HK-2 cells were incubated with SB (50 *μ*M) or vehicle for 1 h and then incubated with 250 mM H_2_O_2_ or vehicle for 24 h and then incubated with the CCK-8 solution for 3 h at 37°C. The maximum absorption of the strong orange CCK-8 formazan was 450 nm. The viability of HK-2 cells was quantified by optical density (OD) measurements using a spectrophotometer. Treatment with H_2_O_2_ markedly increased cellar ROS levels and decreased cell viability, while treatment with SB decreased cellar ROS levels and improved cell viability. In contrast, the supportive effect of SB was blunted in cells transfected with siNrf2. (c, d) Representative immunoblots and statistical data of (c) phosphorylated and total Akt (56 KDa) and (d) nuclear-Nrf2 (68 KDa) at 24 h after treatment with H_2_O_2_ or vehicle. SB could activate Nrf2 by inducing the phosphorylation of Akt, which was inhibited by wortmannin. SFN could activate Nrf2. siNrf2 suppressed the expression of nuclear-Nrf2. Note that SB protected the kidneys from oxidative stress through the PI3K/Akt/Nrf2 pathway. ^*∗∗*^ *p* < 0.01 versus the H_2_O_2_ group; ^##^ *p* < 0.01 versus the SB + H_2_O_2_ group; and ^&&^ *p* < 0.01 versus the siCTRL + SB + H_2_O_2_ group; *n* = 8. The values shown are the mean ± SD.

The effect of SB on cellular ROS levels and the subsequent cell viability induced by H_2_O_2_ were examined using human proximal tubule (HK-2) cells. The cellular ROS production induced by H_2_O_2_ in the SB + H_2_O_2_ group was significantly lower than that in the H_2_O_2_ group (relative fluorescence units, 114.67 ± 4.51 versus 168.00 ± 17.09, *p* < 0.01) (Figure 4(a)). Furthermore, the subsequent cell viability induced by H_2_O_2_ in the SB + H_2_O_2_ group was significantly higher than that in the H_2_O_2_ group (94.33 ± 4.04 versus 50.67 ± 3.06, *p* < 0.01) (Figure 4(b)).

To determine whether the PI3K/Akt/Nrf2 pathway was involved in antioxidative and cell-protective effects of SB, a specific inhibitor of PI3K (wortmannin) was used to abolish the effects of SB. HK-2 cells were transfected with siNrf2 or siCTRL for 6 h and then treated with SB and H_2_O_2_. The results showed that the knockdown of Nrf2 partially reversed the inhibitory effects of SB on the H_2_O_2_-induced ROS production and cellular damage (Figures 4(a) and 4(b)). Western blot analysis showed that SB significantly increased the expression of p-Akt, nuclear-Nrf2 at 24 h after treatment, which was abolished by wortmannin. siNrf2 significantly reversed SB-induced expression of nuclear-Nrf2 and had no an effect on the p-Akt level (Figures 4(c) and 4(d)). The above results suggested that SB-induced antioxidative and cell-protective activity was mediated via the PI3K/Akt/Nrf2 pathway.

## 4. Discussion

SB, the most abundant and bioactive ingredient in Danshen, is widely applied in the treatment of various cardiovascular diseases [18–20], neural diseases [21, 22], and liver diseases [23, 24] and can prevent tubular epithelial-to-mesenchymal transition in the fibrotic kidney induced by HgCl_2_ as well as HK-2 cells triggered by TGF-beta1 [25]. However, little is known about its renoprotection against CI-AKI. In the present study, treatment with SB markedly suppressed the deterioration of renal function in a CI-AKI rat model. The experiment revealed that SB rescued renal tubular injury and prevented apoptosis of renal tubular cells induced by iohexol. The study shows that SB can be effective in preventing kidney damage induced by CM.

Oxidative stress plays a major role in many pathophysiological conditions from a variety of ischaemic/reperfusion or toxic injury [26–30]. Moreover, antioxidants (e.g.,* N*-acetylcysteine [31, 32], statins [33, 34], tocopherol [6, 35], ascorbic acid [36],* Phyllanthus emblica* extract [37], and green tea extract [38]) have been successfully used in experimental or clinical trials for renoprotection against CM. Therefore, oxidative stress is involved in the development and progression of CI-AKI. Previous studies with SB have suggested that it possess the organ-protective capabilities by scavenging ROS [39]. In the present study, SB inhibited the development of oxidative stress in kidneys of CI-AKI rats and H_2_O_2_-induced HK-2 cells damage. The above results showed that SB exerted antioxidative effects to protect renal tubular cells against CI-AKI.

The CI-AKI model in our study used rats with a form of CKD; this model involves dehydration and a nonpharmacological procedure, which was applied to better mimic the clinical disease [14]. We further explored the underlying mechanisms of SB's renoprotection against CI-AKI. Wang et al. reported that SB pretreatment provided significant protection against arsenic trioxide-induced H9c2 cardiomyocyte death correlated with the activation of the PI3K/Akt pathway [40]. Previous research has demonstrated that the PI3K/Akt pathway plays a critical role in modulating Nrf2/HO-1 protein expression as an upstream signaling molecule [41]. In this study, we investigated whether the mechanisms of renoprotection conferred by SB involved activation of the PI3K/Akt/Nrf2 pathway. We found that SB induced Akt phosphorylation and activation of Nrf2, decreased the levels of oxidative stress* in vivo* and* in vitro*, and attenuated the levels of renal dysfunction, tubular damage, and tubular cell apoptosis. These above effects were abolished by treatment with wortmannin (a specific inhibitor of PI3K). The antioxidant and cell-protective effects of SB were reversed by the knockdown of Nrf2* in vitro*. Moreover, an activator of Nrf2, sulforaphane, decreased the levels of oxidative stress as well as attenuated the levels of renal dysfunction, tubular damage, and tubular cell apoptosis in the CI-AKI rat model mimicking the effect of SB. The above results showed that the renoprotection afforded by SB was mediated via the PI3K/Akt/Nrf2 signaling pathway.

Lots of evidence suggests that increased oxidative stress and altered apoptosis are responsible for the pathogenesis of CI-AKI. Oxidative stress in the kidneys is responsible for CI-AKI because it causes significant harmful effects to cellular function, injury to mitochondria, release of apoptosis-inducing factors, activation of the caspase cascade, and damage to nucleic acids, proteins, and membrane lipids [15]. Cytochrome c in mitochondria released into the cytosol can induce apoptosis by activation of downstream caspases. Mitochondrial DNA is a target for oxidative damage and, when damaged, exacerbates oxidative stress by reducing the production of critical proteins for electron transport. This can result in a vicious circle of ROS production and damage to the kidneys that eventually leads to either renal tubular cells injury or apoptosis [42]. Moreover, ROS generation in medullary tubular is also involved in regulating microcirculation through its effects on nitric oxide levels [43].

This experimental model has revealed that SB is associated with renoprotection, which is an encouraging result with potential clinical applications. Moreover, we found that renoprotection was dependent on activation of Nrf2. Therefore, further studies in this regard will be helpful in exploring this new possible therapeutic agent. We speculate that other Nrf2 activators (e.g., sulforaphane [44]; tBHQ [45]; plumbagin [46]; RS9 [47];* trans*-Coniferylaldehyde [48]; and oltipraz [49]) may be promising and effective renoprotective agents due to their antioxidative effects. This study on the PI3K/Akt/Nrf2 pathway of SB not only uncovered its underlying molecular mechanisms but also opens up a new direction for research for the prevention of CI-AKI by targeting the regulation of Nrf2 activity. Therefore, randomized controlled clinical trials will be necessary to explore the effects of SB or Nrf2 activators on clinical outcomes in patients with renal injury and to demonstrate SB or Nrf2 activators may be a novel renoprotective strategy.

## 5. Conclusion

SB can protect against CI-AKI* in vivo* and* in vitro*. The underlying mechanism by which SB protects against CI-AKI is suppression of oxidative stress through the PI3K/Akt/Nrf2 pathway.

## Abbreviations

CI-AKI: Contrast-induced acute kidney injury
CM: Contrast medium
Scr: Serum creatinine
ROS: Reactive oxygen species
SB: Salvianolic acid B
Nrf2: Nuclear factor erythroid 2-related factor 2
HO-1: Heme oxygenase 1
GCLC: Glutamate-l-cysteine ligase catalytic subunit
NQO1: Quinone oxidoreductase
SFN: Sulforaphane
NE: Nephrectomy
BUN: Blood urea nitrogen
HE: Haematoxylin and eosin
TUNEL: Terminal deoxynucleotidyl transferase-mediated dUTP nick-end labeling
PBS: Phosphate-buffered saline
8-OHdG: 8-Hydroxydeoxyguanosine
MDA: Malondialdehyde.
